# S 38093, a histamine H_3_ antagonist/inverse agonist, promotes hippocampal neurogenesis and improves context discrimination task in aged mice

**DOI:** 10.1038/srep42946

**Published:** 2017-02-20

**Authors:** Jean-Philippe Guilloux, Benjamin A. Samuels, Indira Mendez-David, Alice Hu, Marjorie Levinstein, Charlène Faye, Maryam Mekiri, Elisabeth Mocaer, Alain M. Gardier, René Hen, Aurore Sors, Denis J. David

**Affiliations:** 1 CESP/UMR-S1178, Univ. Paris-Sud, Fac. Pharmacie, INSERM, Université Paris-Saclay, Chatenay-Malabry, France; 2 Behavioral and Systems Neuroscience Area, Department of Psychology, Rutgers The State University of New Jersey, Piscataway, NJ, USA; 3 Departments of Neuroscience and Psychiatry, Columbia University, New York, USA; 4 Pôle d’Innovation Thérapeutique Neuropsychiatrie Servier, Suresnes, France; 5 Department of Integrative Neuroscience, New York State Psychiatric Institute, New York, USA

## Abstract

Strategies designed to increase adult hippocampal neurogenesis (AHN) may have therapeutic potential for reversing memory impairments. H_3_ receptor antagonists/inverse agonists also may be useful for treating cognitive deficits. However, it remains unclear whether these ligands have effects on AHN. The present study aimed to investigate the effects of a 28-day treatment with S 38093, a novel brain-penetrant antagonist/inverse agonist of H_3_ receptors, on AHN (proliferation, maturation and survival) in 3-month-old and in aged 16-month-old mice. In addition, the effects of S 38093 treatment on 7-month-old APPSWE Tg2576 transgenic mice, a model of Alzheimer’s disease, were also assessed. In all tested models, chronic treatment with S 38093 stimulated all steps of AHN. In aged animals, S 38093 induced a reversal of age-dependent effects on hippocampal brain-derived neurotrophic factor (BDNF) BDNF-IX, BDNF-IV and BDNF-I transcripts and increased vascular endothelial growth factor (VEGF) expression. Finally, the effects of chronic administration of S 38093 were assessed on a neurogenesis-dependent “context discrimination (CS) test” in aged mice. While ageing altered mouse CS, chronic S 38093 treatment significantly improved CS. Taken together, these results provide evidence that chronic S 38093 treatment increases adult hippocampal neurogenesis and may provide an innovative strategy to improve age-associated cognitive deficits.

Adult neurogenesis, a process in which new neurons are continuously generated in adulthood, occurs in the subgranular zone (SGZ) of the hippocampal dentate gyrus (DG) of adult mammalian brains^1^,^2^,^3^,^4^. Although the precise role of this neuronal turnover in the adult brain remains unknown, several studies suggest a relationship between neurogenesis and hippocampal-dependent cognitive functions. Indeed, suppression of neurogenesis in the DG by X-ray irradiation or treatment with an antimitotic agent impaired hippocampus-dependent learning^5^,^6^. Furthermore, brain-derived neurotrophic factor (BDNF) enhances hippocampal neurogenesis and improves learning and mental performance^7^.

Recent studies have implicated adult-born hippocampal neurons in context discrimination, a process by which similar experiences or events are transformed into discrete, non-overlapping representations^8^,^9^. Cognitive deficits consistent with impaired context discrimination in humans during normal aging and in patients with mild cognitive impairment have been also described^10^. Thus, strategies that specifically increase adult hippocampal neurogenesis may have therapeutic potential for reversing hippocampal memory impairments such as those seen during normal ageing or in diseases with cognitive decline^8^,^11^,^12^.

Modulation of the histaminergic H_3_ subtype receptor has been proposed for the treatment of cognitive deficits observed in Alzheimer’s disease (AD)^13^,^14^,^15^. The H_3_ subtype receptor, which is widely expressed in the brain, is, among the 4 types of histaminergic receptors, particularly involved in cognitive processes. The H_3_ receptor has a mostly pre-synaptic localization on histaminergic neurons and its activation leads to the inhibition of the synthesis and release of histamine^16^. In addition, activation of postsynaptic H_3_ heteroreceptors on heterologous nerve endings likely decreases the release of neurotransmitters such as acetylcholine, serotonin, noradrenaline and dopamine^17^,^18^. Recently, S 38093, an inverse agonist/antagonist of H_3_ receptors^19^, was found to be active at a mean pharmacological dose of 0.3–1 mg/kg p.o./i.p. in animal behavioral tests of working memory (Morris water maze in rats; spontaneous alternation and concurrent serial alternation tests in mice; delayed matching to sample in aged monkeys) and episodic-like memory (social and object recognition tests in rats; contextual discrimination task in mice)^20^,^21^. S 38093 also improves attention, executive functioning, and cognitive flexibility in MPTP-treated monkeys. Moreover, in line with its H_3_ antagonist/inverse agonist properties, S 38093 dose-dependently increases extracellular histamine levels in the prefrontal cortex and facilitates cholinergic transmission in the prefrontal cortex and hippocampus of rats after both acute and chronic administrations^20^. While these data indicate that S 38093 has pro-attentional and pro-cognitive properties, interestingly they may also suggest that S 38093 can enhance adult hippocampal neurogenesis. Substantial evidence indicates that cholinergic neurotransmission regulates adult hippocampal neurogenesis^22^. Furthermore, depletion of cholinergic input to the neurogenic niche during the aging process may contribute to spatial memory deficit^23^.

In this study we investigated the effects of chronic S 38093 administration (0.3, 1 and 3 mg/kg/day p.o., 28 days) on hippocampal neurogenesis (proliferation, maturation and survival) in different mouse models, in young adult, aged and AD transgenic mice (APPSWE Tg2576). In parallel, biological markers involved in both hippocampal neurogenesis and Alzheimer’s disease, such as BDNF, VEGFA and IGF-1, were evaluated in aged mice. Finally, the effect of chronic administration of S 38093 (0.3 and 3 mg/kg/day p.o., 28 days) was assessed on a neurogenesis-dependent task as a model of “context discrimination” in aged mice.

## Results

Detailed statistical results are provided in Supplementary Table 1, 2, 3, 4, 5, 6 and 7.

### Chronic S 38093 administration enhances hippocampal neurogenesis in young adult 129/SvEvTac mice

**Figure 1:**
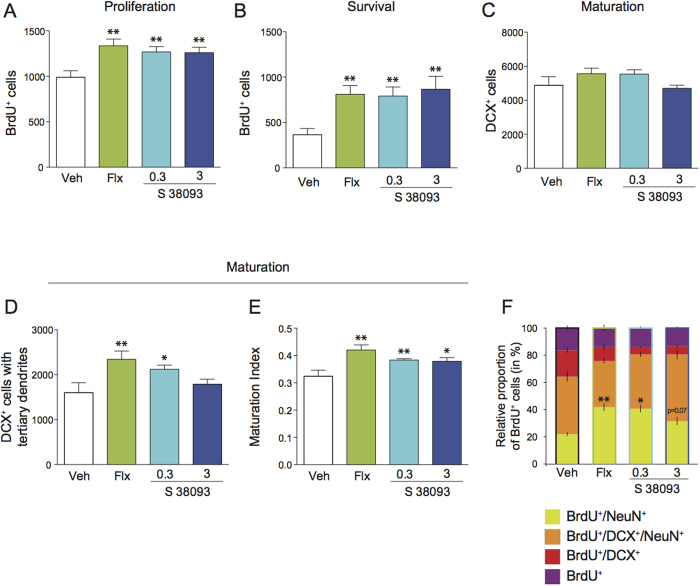
Chronic S 38093 administration enhances adult hippocampal neurogenesis in the dentate gyrus of young adult 129/SvEvTac mice. The effects of chronic administration of S 38093 (0.3 and 3 mg/kg/day p.o., 28 days) and fluoxetine (18 mg/kg/day p.o., 28 days) on cell proliferation (**A**), survival (**B**) and maturation (**C**–**F**) were compared to those of vehicle (purified water). Cell proliferation (**A**) and survival (**B**) were expressed by the number of BrdU^+^ cells. Maturation was represented by the total number of DCX^+^ cells (**C**), the number of DCX^+^ cells with tertiary dendrites (**D**) and the maturation index of newborn granule cells (**E**). The fate of proliferating cells was characterized using co-localization of BrdU^+^ cells with markers of neuronal maturation (DCX) or mature neurons (NeuN) (**F**). Data are expressed as mean ± SEM. A one-way ANOVA was applied to the data followed by Fisher’s PLSD post hoc analysis as appropriate. *p < 0.05, **p < 0.01 compared to vehicle-treated mice (n = 5–10 mice of 3 months old per group).

Chronic S 38093 administration (0.3 and 3 mg/kg/d p.o., 28 days) significantly increased proliferation of progenitors in the DG of hippocampus in young adult mice, with similar effects to those obtained with chronic fluoxetine administration (18 mg/kg/d p.o., 28 days) (+28.1%,+27.5% and +35.0% of BrdU+ cells in comparison to vehicle group, respectively (p < 0.01) (Fig. 1A). The same dosing regimen of S 38093 and fluoxetine also significantly increased the survival of newborn cells: +117.1%, +137.5% and +122.4% of BrdU-positive cells in comparison to vehicle group, respectively (p < 0.01) (Fig. 1B). The total number of DCX^+^ cells was unchanged after either chronic S 38093 or fluoxetine administration (Fig. 1C), however chronic S 38093 (0.3 mg/kg/d) or fluoxetine treatment significantly increased the number of DCX^+^ cells with tertiary dendrites (p < 0.05 and p < 0.01, respectively, Fig. 1D). The maturation index was increased for both doses of S 38093 (0.3 mg/kg/d: p < 0.01; 3 mg/kg/d: p < 0.05, Fig. 1E) and fluoxetine (p < 0.01). Interestingly, S 38093 as fluoxetine, increased proliferation, survival and maturation not only at the ventral but also at the dorsal part of the DG in 3-month old 129/SvEVTac mice (Supplementary Figs S2A,B,C,D,E,F). Then, the relative “maturity” of BrdU+ cells was investigated by determining whether they expressed DCX and/or NeuN (Fig. 1F). As expected, the number of immature BrdU^+^ granule cells (BrdU^+^/DCX^+^/NeuN^−^) decreased in the fluoxetine group, indicating that the immature cells mature out of the DCX stage more quickly than in the vehicle group (p < 0.01). Both doses of S 38093 also enhanced maturation of newborn neurons, as the proportion of BrdU^+^/NeuN^+^ cells in these groups increased from 21.9% in vehicle treated animals to 40.9% and 31.3% for S 38093 at 0.3 (p < 0.01) and 3 mg/kg/d (p = 0.07). The number of BrdU^+^/DCX^−^/NeuN^−^ cells, which likely represents astrocytes, did not change across groups (Supplementary Figs S4, S5 and S6, Supplemental Movie).

### Chronic S 38093 administration enhances hippocampal neurogenesis and facilitates maturation of newborn neurons in aged C57Bl/6JRj mice and in the APPSWE model of Alzheimer’s disease

**Figure 2:**
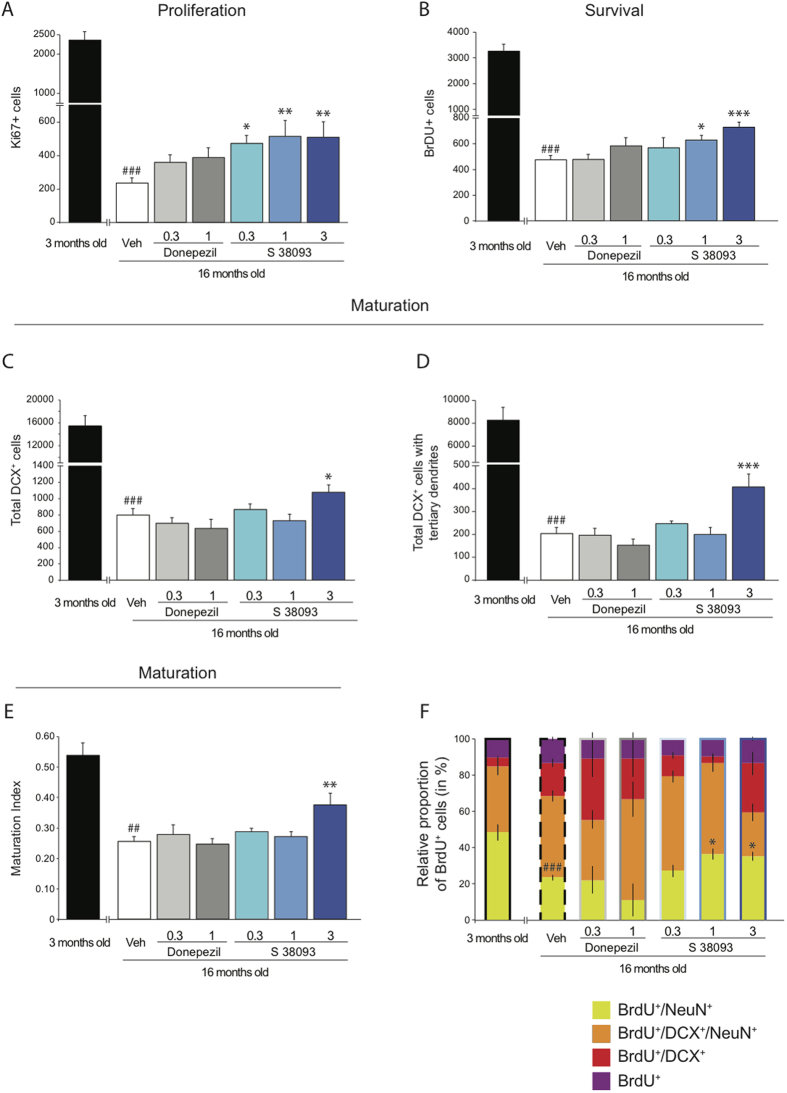
Chronic S 38093 administration enhances adult hippocampal neurogenesis in the dentate gyrus old C57Bl/6JRj mice. The effects of chronic administration of S 38093 (0.3, 1 and 3 mg/kg/day p.o., 28 days) and donepezil (0.1 and 1 mg/kg/day p.o., 28 days) on cell proliferation (**A**), survival (**B**), maturation (**C**–**F**) were compared to those of vehicle (purified water). Cell proliferation (**A**) and survival (**B**) were expressed by the number of BrdU^+^ cells. Maturation was represented by the total number of DCX^+^ cells (**C**), the number of DCX^+^ cells with tertiary dendrites (**D**) and the maturation index of newborn granule cells (**E**). The fate of proliferating cells was characterized using co-localization of BrdU^+^ cells with markers of neuronal maturation (DCX) or mature neurons (NeuN) (**F**). Data are expressed as mean ± SEM. A one-way ANOVA was applied to the data followed by Fisher’s PLSD post hoc analysis as appropriate. *p < 0.05, **p < 0.01, ***p < 0.0001 for effects of S 38093 compared to vehicle; ^##^p < 0.01, ^###^p < 0.0001 for effects of aging in old compared to young vehicle-treated mice (n = 4–8 mice of 3 or 16 months old per group).

Aging strongly affects cell proliferation of progenitors (p < 0.001, Fig. 2A), survival (p < 0.001, Fig. 2B) and maturation of newborn neurons (p < 0.001, Fig. 2C,D). There is a 2-fold decrease in the maturation index (p < 0.01, Fig. 2E). Donepezil at all doses tested had no effect on neurogenesis. Similar to the study in young mice, S 38093 (0.3, 1 and/or 3 mg/kg) significantly increased cell proliferation, survival, and maturation in the DG of hippocampus in aged mice relative to vehicle. (Fig. 2A–E). As expected, the number of immature BrdU^+^ granule cells (BrdU^+^/DCX^+^/NeuN^−^) decreased in 16-month-old aged mice compared to 3-month-old young adult mice (p < 0.01) (Fig. 2F). High doses of S 38093 also increased the maturation of newborn neurons, as the proportion of BrdU^+^/NeuN^+^ cells in these groups increased (1 and 3 mg/kg/d: p < 0.05). The proportion of BrdU^+^/NeuN^+^ cells in this group decreased from 48.5% in 3-month old mice to 23.7% in 16-month old mice (p < 0.01). The number of BrdU^+^/DCX^−^/NeuN^−^ cells, which likely represents astrocytes, did not change across groups. Donepezil did not affect the fate of the proliferating cells compared to vehicle-treated groups.

The neurogenic effects of chronic administration of S 38093 (3 mg/kg/d p.o., 28 days) were next evaluated in 7-month-old AD transgenic mice (APPSWE model). A two-way ANOVA revealed a significant effect of treatment for all the different analyzed parameters for hippocampal neurogenesis. S 38093 increased cell proliferation and had a strong effect on cell survival (+117%) compared to vehicle group within the same genotype, p < 0.05) (Table 1). A significant effect of treatment for all the different parameters for maturation was also observed. The total number of DCX^+^ cells with tertiary dendrites and the maturation index were also significantly increased after S 38093 in both genotypes (p < 0.05 and p < 0.01, respectively). Interestingly, chronic S 38093 administration also increased the dendritic intersections in both genotypes (one-way ANOVA with repeated measure, p < 0.01), with a significant effect from 50 to 80 in APPSWE^TG^ mice only (p < 0.05). A trend for an increase in dendritic length after S 38093 was also observed in both genotypes (Supplementary Figs S3A,B,C,D,E and S7).

### Chronic S 38093 administration modulates hippocampal expression of genes related to neurogenesis and hippocampal function

**Figure 3:**
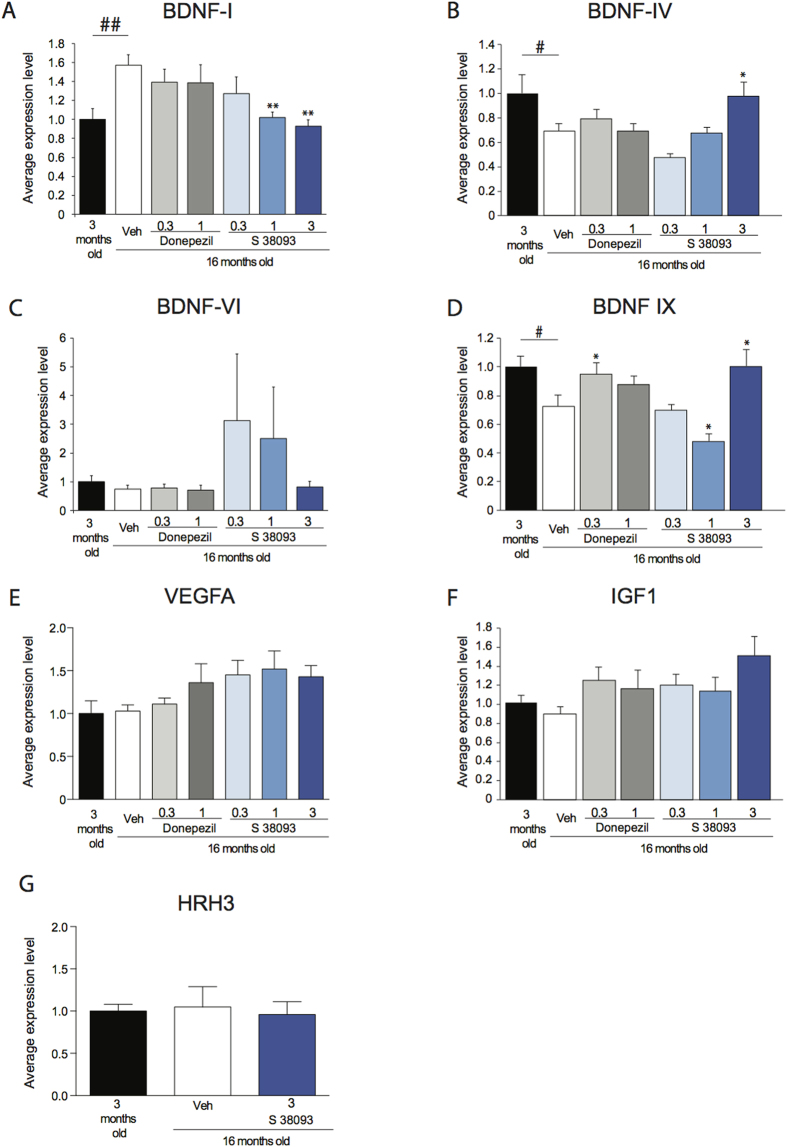
Transcriptional effects in hippocampus of aging and chronic S 38093 administration in old C57Bl/6JRj mice. The effects of aging and of chronic treatment with S 38093 (0.3, 1 and 3 mg/kg/day p.o., 28 days) donepezil (0.1 and 1 mg/kg/day p.o., 28 days) on hippocampal activity-dependent exons of BDNF (**A**–**C**), total BDNF (**D**), VEGFA (**E**), IGF1 (**F**) and H_3_R (**G**) gene expression were quantified. Data are expressed as mean ± SEM. *p < 0.05 and **p < 0.01 for effects of S 38093 compared to vehicle. A one-way ANOVA was applied to the data followed by Fisher’s PLSD post hoc analysis as appropriate. ^#^p < 0.05, ^##^p < 0.01 for effects of aging in old compared to young vehicle-treated mice (n = 5–7 mice of 16 months old per group).

In aged mice, a decrease in the expression of whole BDNF transcripts (BDNF-IX, −28%, p < 0.05) and BDNF-IV (−40%, p < 0.05) activity-dependent transcripts and an increase in BDNF-I activity-dependent transcripts (+57%, p < 0.01) expression were observed compared to young mice (Fig. 3A,B). No significant age effect was observed on BDNF-VI, VEGF and IGF-1 transcripts (Fig. 3C–F).

In aged mice, chronic administration of S 38093 (1 and/or 3 mg/kg/day p.o., 28 days) reversed this age-dependent decrease in BDNF-IX, BDNF-IV and BDNF-I transcripts. Donepezil only reversed the age-dependent decrease in BDNF-IX transcripts at 0.3 mg/kg/d. In addition, S 38093 at three tested doses (0.3, 1 and 3 mg/kg/d) increased VEGF transcripts compared to vehicle-aged group. However, this increase was only significant at a dose of 1 mg/kg/d (p < 0.01).

We finally evaluated whether or not changes in the levels of histamine H_3_ receptor transcripts in the mice hippocampus could be responsible for the difference of sensitivity of H_3_ receptor to S 38093 in young *versus* aged mice. Results showed that there is no age or treatment effect on the H_3_ receptor transcripts expression in the hippocampus. Indeed, the level of H_3_ transcripts of aged mice treated or not with the highest dose of S 38093 (3 mg/kg/day) was not statistically different from that of 3-months old animals (Fig. 3G).

### Chronic S 38093 administration increases context discrimination in aged C57Bl/6JRj mice

**Figure 4:**
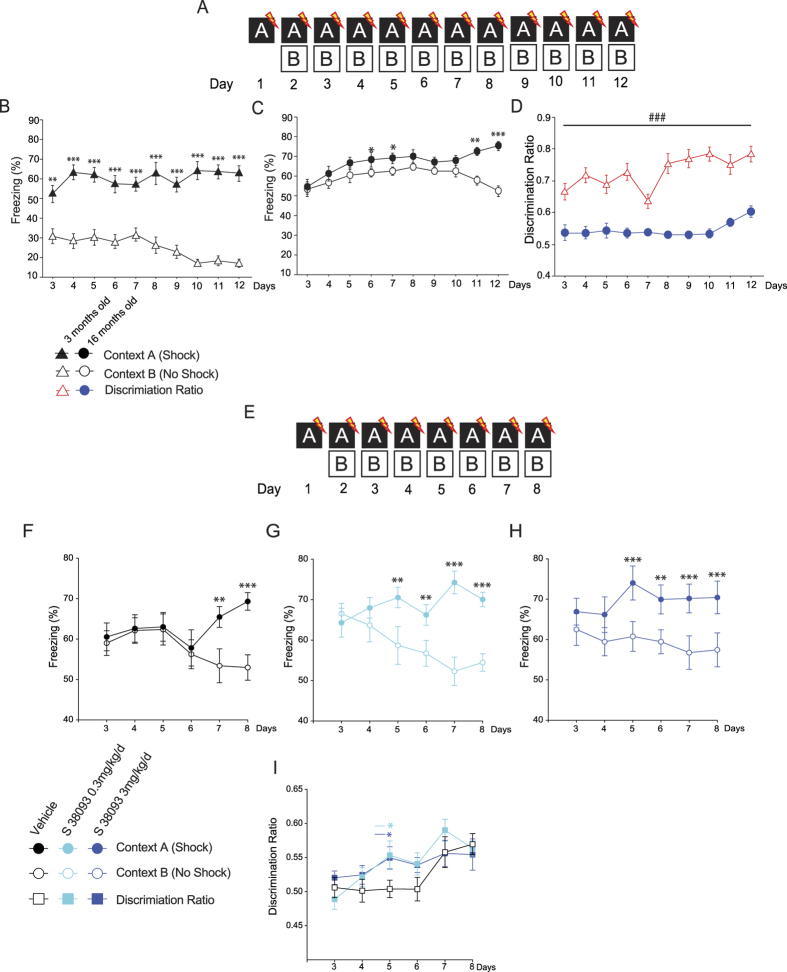
Chronic S 38093 administration improves context discrimination in old C57Bl/6JRj mice. Schematic of the Context discrimination task protocol used to evaluate the effects of age (**A**). Freezing duration in percentage between contexts A and B in 3-month-old animals **(B)** and in 16-month-old animals **(C)**. Discrimination ratio in young and old mice **(D)** (n = 12–15 young adult or aged mice per group). Schematic of the Context discrimination task protocol used to evaluate the effects of S 38093, a histamine H_3_ antagonist/inverse agonist (**E**). 28 days after the start of treatment, on “day 1” the animals were only exposed to shock context A and on every subsequent day animals were exposed to shock context A then the similar non-shock context B in that order. Context discrimination was measured as the percentage of freezing behavior in both contexts for vehicle (purified water) (**F**), S 38093 0.3 mg/kg/day p.o. (**G**) and S 38093 3 mg/kg/d p.o. (**H**) groups. Discrimination ratios were then calculated across days for each treatment group (**I**). A two-way ANOVA with repeated measures was applied to the data. Significant main interactions were followed by Fisher’s PLSD post hoc analysis. *p < 0.05, **p < 0.01, ***p < 0.0001 for effects of age (Fig. 4B,C,D) or **p < 0.01, ***p < 0.0001 for effects of treatment (Fig. 4F,G,H) (n = 12–15 young adult or aged mice per group). ^###^p < 0.001 for comparisons between young adult and old mice. ^§^p < 0.05 planned comparison between S 38093 (0.3 or 3 mg/kg/d) and vehicle group for discrimination ratios (Fig. 4I).

Aging significantly alters “context discrimination” in a contextual fear conditioning paradigm as previously described^24^. We confirmed these findings using a context discrimination task (Fig. 4A,B,C and D). We next assessed whether S 38093 could reverse these age-dependent effects. A two-way ANOVA with repeated measured revealed that the difference in the percent time spent freezing between the two similar contexts was significantly different beginning on day 7 for the vehicle group (day 7: p < 0.01; day 8: p < 0.001, Fig. 4F). After a low dose of a chronic S 38093 administration (0.3 mg/kg/d p.o., 28 days), animals displayed a difference in the percent time freezing between the two contexts starting at day 5 (0.3 mg/kg/d: day 5–6: p < 0.01; day 7–8: p < 0.001, Fig. 4G). For the highest dose tested (3 mg/kg/d p.o., 28 days) a significant effect of treatment was observed (p < 0.001, Fig. 4H). Overall, S 38093-treated mice began discriminating between the two similar contexts earlier than the vehicle group. Therefore, the S 38093-treated mice showed an increase in their discrimination ratio as early as day 5 compared to vehicle-treated animals (p < 0.05, Fig. 4I).

## Discussion

Strategies that increase adult hippocampal neurogenesis have been researched for their therapeutic potential as antidepressants^25^. Here, we suggest that such strategies may also be useful for treating hippocampal memory impairments as those seen during normal ageing^8^ or for preserving cognitive capacity^26^. This is the first study suggesting that H_3_ receptor antagonists/inverse agonists may be a novel strategy for increasing adult hippocampal neurogenesis, and thus H_3_ may be a new target for treating depression^27^ or cognitive deficits^13^,^14^,^15^. Using a novel brain-penetrant antagonist and inverse agonist of both rodent and human H_3_ receptors^20^,^21^, we found that chronic S 38093 treatment at all tested doses increased both proliferation and survival of immature neurons in the adult DG of the hippocampus of 129SvEv mice (Fig. 1), a well validated strain to investigate pro-neurogenic activity of compounds^28^. Moreover, we demonstrated that chronic low dose of S 38093 stimulates maturation of immature granule cells. A larger fraction of DCX^+^ cells possessed tertiary dendrites after chronic S 38093 treatment, suggesting a more complex dendritic arborization. Overall, newborn neurons underwent an accelerated maturation after chronic S 38093 treatment, as shown by the increased proportion of newborn cells that ceased to express the immature neuronal marker DCX. Interestingly, the H_3_ receptor antagonist/inverse agonist produced similar neurogenic effects as chronic fluoxetine^29^. In the hippocampus, there is very low H_3_ mRNA expression in the CA1 field of Ammon’s Horn and moderate expression in the pyramidal cell layer of the CA3 field. In the DG, intense labeling is found in the subgranular zone, with lower signals in the granular layer^30^. [^14^C]-S 38093-2 binding has been observed in brain areas where H_3_ receptors are present including the hippocampal formation (DG and subiculum)^21^. Moreover, we previously demonstrated that S 38093 facilitated cholinergic transmission in the prefrontal cortex and the hippocampus of rats after administration^20^,^21^. The hippocampus receives abundant cholinergic innervation and ACh plays an important role in learning but can also regulate adult hippocampal neurogenesis^31^. More specifically, changes in forebrain ACh levels primarily influence the proliferation and/or the short-term survival of adult born neurons through activation of muscarinic receptor subtypes M_1_ and M_4_ located on the newly born cells. We cannot rule out that the increase in adult hippocampal neurogenesis may have also been induced via increases in monoamine levels. Serotonin, dopamine, and noradrenaline are all involved in different steps of adult hippocampal neurogenesis^22^.

Since the pro-neurogenic effects of S 38093 appeared promising in adult 129SvEv mice, we also evaluated the consequences of a chronic treatment in aged 16-month old mice. Normal rodent aging is associated with attenuation of hippocampal neurogenesis^3^,^32^ (Fig. 2), and this age-dependent reduction in adult neurogenesis may contribute to cognitive decline^26^. In our study, we first confirmed that ageing induced a 5-fold decrease in cell proliferation, cell survival, maturation, and dendritic arborization of neurons in the DG of the mouse hippocampus. We then looked at the relative “maturity” of BrdU^+^/NeuN^+^ cells based on whether or not they expressed DCX in aged mice. We found an increase in the production of immature neurons and a decrease in the number of newly formed adult neurons. Interestingly, unlike donepezil, a compound used to treat Alzheimer’s disease, chronic treatment with S 38093 increased cell proliferation (0.3 to 3 mg/kg/day) and survival (1 and 3 mg/kg/day) in the DG of the hippocampus of aged mice. We also observed for the high dose (3 mg/kg/day) an increase in the maturation of neurons, the complexity of their dendritic arborizations, and an increase in the number of newly formed adult neurons. If chronic S 38093 did not fully restore the age-dependent deficit in neurogenesis, it showed that stem cell reactivity is still occurring during aging. Since the lowest dose (0.3 mg/kg/day) was the most efficient to stimulate adult hippocampal neurogenesis in young adult mice (3 month-old) and the highest dose (3 mg/kg) in aged animals (16-month-old mice), we evaluated whether changes in H_3_ receptor transcripts could highlights the difference of sensitivity of H_3_ receptor to S 38093 during aging. We confirmed previous results^33^ showing that H_3_ receptor transcripts levels and functionality is unlikely affected during aging.

It is likely that the S 38093-induced effects on dendritic morphology, maturation, and synaptic plasticity are mediated by neurotrophic factors. Indeed, growth factors, including brain-derived neurotrophic factor (BDNF) and vascular endothelial growth factor (VEGF) are upregulated after chronic S 38093 treatment (Fig. 3). The observed variations of expression of BDNF-IX (total BDNF) and BDNF-IV (activity-dependent transcript) induced by either aging or chronic S 38093 administration (3 mg/kg) are associated with changes in various steps of neurogenesis and this association has been previously observed in the literature^34^,^35^. While restoring baseline levels of BDNF is of therapeutic interest, it is the ability to induce activity-dependent BDNF in response to a stimulus that can enhance neuronal survival and plasticity. In the DG, blockade of histamine H_3_ receptors located on perforant path terminals with chronic S 38093 could prevent the inhibition the release of glutamate^36^. Indeed, glutamate signaling is known to induce expression of neurotrophic factors, such as brain-derived neurotrophic factor (BDNF)^22^,^37^. Furthermore, decreases in VEGF levels are found in the hippocampus of aging patients^38^ and in rodents^39^, and chronic VEGF administration ameliorates memory impairments in an APP transgenic mouse model of Alzheimer’s disease^40^. In this study, while no effect of age on VEGF expression was observed, chronic S 38093 administration enhanced VEGF levels in the hippocampus of aged mice.

In this study, we found that chronic S 38093 treatment in aged animals increased the proportion of BrdU^+^/NeuN^+^/DCX^+^ cells (Fig. 2E). These young neurons combined with an increase in dendritic complexity make a major contribution to ACSF–LTP^29^, which may improve cognition. By contrast, deficits in adult hippocampal neurogenesis may impact learning. The DG is a subregion of the hippocampus proposed to play a role in context discrimination, a process by which similar experiences or events are transformed into non-overlapping representations^9^,^41^. Sahay and colleagues suggested that strategies designed to specifically increase adult hippocampal neurogenesis, by targeting cell death of adult-born neurons or other means, may have therapeutic potential for reversing impairments in context discrimination such as that seen during normal aging^8^. Given that chronic S 38093 increased adult hippocampal neurogenesis, we finally explored whether it could also be used to treat cognitive decline^8^,^42^ by assessing the effects of administration in a context discrimination task. As expected, young mice, which have significantly higher levels of neurogenesis, showed context discrimination by day 3 while aged mice did not show discrimination until Day 11. In humans context discrimination has been shown to decline with both normal aging, and in patients with Mild Cognitive Impairment (MCI), and is thought to be impaired in other psychiatric and neurological conditions including Alzheimer’s disease and anxiety-related disorders^43^. In fact, impaired contextual fear discrimination may result in a bias to encode ambiguous cues as threatening and may underlie the excessive generalization observed in posttraumatic stress disorder and panic disorder. Mice treated with either the low (0.3 mg/kg/day) or high dose (3 mg/kg/day) of S 39093 showed significant discrimination by Day 5, while vehicle treated aged mice did not show discrimination until Day 7.

Taken together, this study provides evidence that chronic treatment with a S 38093, a novel brain-penetrant antagonist/inverse agonist of H_3_ receptors, results in a normalization of age-dependent behavioral decline possibly through the release of growth factors (e.g., VEGF and BDNF), which in turn could underlie the increased proliferation of neural progenitors and facilitated maturation of young hippocampal neurons not only in adult animals but also in aged mice and AD mouse model.

## Methods

### Animals

#### Neurogenesis study

##### Study in young mice

Eighty adult (8 weeks old at the start of the protocol) male mice of 129/SvEv Tac strain (Taconic Farms, Denmark) were used for the neurogenesis study.

##### Study in aged mice

One hundred and five C57Bl/6JRj male mice, 15-month-old (25–30 g, Janvier Farms, France) and twenty 2-month-old mice at the start of the protocol were used for the neurogenesis study in old animals (3-month-old and 16-month-old at the end of the protocol).

##### Study in animal model of Alzheimer’s disease

Neurogenesis was also assessed in thirty transgenic male APPSWE-Tg2576 mice (B6; SJL-Tg(APPSWE)2576Kha; model 1349-RD1, Taconic Farms) and their wild-type littermates. Transgenic mice and their littermates received at 2 months old and were kept at the animal facility until their sacrifice at 8 months old.

Mice were maintained under standard conditions (12/12 h light/dark cycle, lights on at 6 AM, 22 ± 1 °C, food and water *ad libitum*, 5 mice/cage). All behavioral testing and gavage were performed during the light cycle.

The protocols involving animals and their care were conducted in conformity with the institutional guidelines that are in compliance with national and international laws and policies (Council directive #87–848, October 19, 1987, Ministère de l’Agriculture et de la Forêt, Service Vétérinaire de la Santé et de la Protection Animale, permissions #92–256B to DJD; NIH Guide for the Care and Use of Laboratory Animals) and in compliance with protocols approved by the Institutional Animal Care and Use Committee (CEE26 authorization 2012–099; Institutional Animal Care and Use Committee of Columbia University and the Research Foundation for Mental Hygiene, Inc.).

#### Behavioral study

Behavioral experiments were conducted in 2 separate cohorts of thirty-nine 15-month-old male C57Bl/6JRj mice (Cohort 1: treatment effect) and fifteen 15-month-old male and fifteen 8-week-old C57Bl/6JRj mice (Cohort 2: age effect) (Charles River, Raleigh, North Carolina, USA) at the start of the protocol.

The mice were housed in groups of two to five per cage and had access to food and water *ad libitum*. The mice were maintained on a 12:12 light/dark schedule and all behavioral testing and gavage was performed during the light cycle. Animal protocols were approved by the Institutional Animal Care and Use Committee of Columbia University and the Research Foundation for Mental Hygiene, Inc. and were in compliance with the NIH Guide for the Care and Use of Laboratory Animals. Care was taken to minimize the both the number of animals used and their suffering.

### Drugs and treatment

#### Drugs

Behavioral and neurogenic effects of S 38093 (Servier, France) were compared to fluoxetine hydrochloride (Anawa Trading, Zurich, Switzerland) or Donepezil hydrochloride (Sigma-Aldrich, St Quentin Fallavier, France).

#### Neurogenesis study

#### Behavioral study

S 38093 (0.3 and 3 mg/kg/day, oral gavage) or its vehicle (purified water) were administered chronically during 28 days, before starting the behavioral experiment (Supplementary Fig. S1D).

### Immunohistochemistry

The effects of chronic treatment with S 38093, a novel brain-penetrant antagonist/inverse agonist of H_3_ receptors, on AHN (proliferation, maturation and survival) were assessed in 3- and 16-month-old mice and in 7-month-old APPSWE Tg2576 transgenic mice, a potential model of Alzheimer’s disease. To this end, after 4 weeks of treatment, animals were anesthetized with ketamine and xylazine (100 mg/ml ketamine; 20 mg/ml xylazine), then perfused transcardially (cold saline for 2 min, followed by 4% cold paraformaldehyde at 4 °C). The brains were then removed and cryoprotected in 30% sucrose and stored at 4 °C. Serial sections (35 μm) were cryosectioned through the entire hippocampus (−1.10 to −3.80 mm relative to Bregma according to Franklin and Paxino’s brain atlas (2008)^44^ and stored in PBS with 0.1% NaN_3_. Since there is a differential contribution of adult hippocampal neurogenesis to cognition and mood along the septotemporal axis of the dentate gyrus, the effects of S 38093 and fluoxetine along the septotemporal axis on proliferation, maturation, survival and neurogenesis were also analyzed (Supplemental Fig. 2). The coordinates used to dissociate the dorsal and ventral hippocampus were based on previous publications^45^,^46^: from −1.10 to −2.50 mm relative to Bregma for the dorsal hippocampus and from −2.50 to −3.80 mm for the ventral hippocampus according to Franklin and Paxino’s brain atlas (2008)^44^.

#### Proliferation study

5-bromo-2-deoxyuridine (BrdU) or Ki67 labeling were used for proliferation study in the DG of young adult mice. BrdU (150 mg/kg i.p.) was administered 2 h before sacrifice and processed as previously described^25^. Briefly, for DAB staining, sections were mounted on slides and boiled in citric acid (pH 6.0) for 5 min, rinsed with PBS. Peroxydase was quenched by 0.3% H_2_O_2_ in 0.1 M TBS. Sections were treated with 0.01% trypsin in Tris/CaCl_2_ for 10 min. Brain sections were incubated for 30 min with 2 N HCl and blocked with 5% NGS. Sections were then incubated overnight at room temperature with anti-mouse BrdU (1:100) (Becton Dickinson, France). After washing with PBS, sections were incubated for 1 hr with secondary antibody (1:200 biotinylated goat anti-mouse, Vector, Burlingame, CA) followed by amplification with an avidin-biotin complex. The staining was visualized with DAB.

In aged and AD transgenic mice (APPSWE Tg2576), proliferation of progenitors was assessed using Ki-67 as a marker for cell division. Ki67, a nuclear protein expressed in all phases of the cell cycle except for G0, can be used as an endogenous marker alternative to BrdU incorporation^47^. Ki-67 immunohistochemistry as described previously^45^. Briefly, sections were washed in PBS, blocked (PBS containing 0.3% triton and 10% NDS) and incubated with primary antibody overnight at 4 °C (Ki67 rabbit, 1:100, Vector, Burlingame, CA). Following washes in PBS, sections were incubated with fluorescence coupled rabbit secondary antibody (Jackson ImmunoResearch, France). Using a BX51 microscope (Olympus, Germany), 1/6 and 1/12 of the hippocampus were counted for BrdU^+^ or KI67^+^ cells respectively by treatment-blind experimenter.

#### Survival study

Young adult, aged and AD transgenic mice (APPSWE Tg2576) mice were administered BrdU (150 mg/kg i.p. twice a day, for 3 days) 4 weeks before sacrifice. We then proceeded as described previously using a similar DAB protocol to count BrdU^+^ cells.

#### Doublecortin (DCX) labelling for maturation index study

The immunohistochemistry protocol was adapted from David *et al*.^25^. For doublecortin staining, the procedure consisted of the following steps: 1 hr incubation in 0.1 M TBS with 0.5% Triton X-100 and 10% normal donkey serum (NDS), followed by goat anti-doublecortin primary antibody (1:100) in TBS/Tx/NDS for 24 hrs at 4 °C. The secondary antibody was biotinylated donkey anti-goat (1:500) in TBS/NDS for 1 hr at room temperature, followed by a 1hr amplification step using an avidin-biotin complex (Vector, USA). DCX-positive (DCX^+^) cells were subcategorized according to their dendritic morphology: DCX^+^ cells and DCX^+^ cells with tertiary (or higher order) dendrites. The maturation index was defined as the ratio of DCX^+^ cells possessing tertiary dendrites to the total number of DCX^+^ cells as described previously^48^. 1/12 of the hippocampus was counted using a BX51 microscope (Olympus, Germany) by treatment-blind experimenter.

#### Immunohistochemistry and confocal imaging for maturation study

Immunohistochemistry was performed in the following steps: 2 h incubation in 1:1 formamide/2xSSC at 65 °C, 5 min rinse in 2xSSC, 30 min incubation in 2 N HCl at 37 °C, and 10 min rinse in 0.1 M boric acid, pH 8.5, 2 h incubation in 0.1 M PBS with 0.3% Triton X-100, and 5% normal donkey serum. Sections were then incubated overnight at 4 °C in primary antibodies for doublecortin (goat 1:500; Santa Cruz Biotechnology, Santa Cruz, CA), bromodeoxyuridine (BrdU; rat; 1:100; Serotec, Oxford, UK) and neuronal-specific nuclear protein (NeuN) (mouse; 1:500; Chemicon, Temecula, CA). Then fluorescent secondary antibodies were used. All secondary antibodies were purchased from Jackson ImmunoResearch (France). Approximately 6 sections per animal and 20–30 BrdU^+^ cells per treatment group were analysed (n = 4–5 animals per conditions), Among the BrdU^+^ cells, the percentage of BrdU^+^/DCX^+^/NeuN^−^, BrdU^+^/DCX^+^/NeuN^+^ and BrdU^+^/DCX^−^/NeuN^+^ cells were evaluated for each treatment group. All cell counting for triple-stained sections were done using a Zeiss LSM 510 confocal microscope (X63 magnification).

### Sholl analysis

For Sholl analysis, DCX^+^ cells with tertiary, relatively untruncated dendritic branches were traced for each 35 μm hippocampal slice using Neurolucida software (MicroBrightField, Williston, VT) on an Olympus BX51 microscope equipped with a motorized stage device and x100 immersion oil objective. DCX immunohistochemistry was done to maximize the labelling of dendrites. Sholl analysis for dendritic complexity was performed using the accompanying software (NeuroExplorer; MicroBrightField, version 10), calculating dendritic complexity including dendritic length and number of intersections (branch points) as described previously^48^.

### Behavioural analysis

#### Contextual Fear Discrimination

The context discrimination task consisted of an 8-day contextual fear discrimination paradigm in which the mice had to learn to distinguish between a fearful shock context and a similar non-shock context. On day 1 the mice were only exposed to the training shock context, and on days 2–8 the mice were exposed to the shock and then non-shock context in that order each day. The mice were run through the context discrimination task starting at 10 am and ending at 2 pm and were then gavaged with vehicle or S 38093 at 4 pm each day. The drug and vehicle were always administered after behavioral testing to avoid possible acute effects of the drug on behavior. Learning was measured by the percent time the mice spent freezing and testing was terminated when percent freezing was consistently significantly different between the two contexts. The mice were tested after 29 days of drug treatment. Conditioning was conducted on one side of a Med-Associates shuttle box (ENV-010MC; 20.3 cm × 15.9 cm × 21.3 cm high) with a clear plexiglass wall, 3 aluminum walls and a stainless steel grid as a floor. Mouse behavior was recorded by digital video cameras mounted above the conditioning chamber. Freezeframe and Freezeview software (Actimetrics, Evanston, IL) were used for recording and analyzing freezing behavior, respectively. In training context A, mice were allowed to habituate in new cages outside the room and were then brought into the room in the new cages. The house fan and house light (CM1820 bulb) were turned on, the stainless steel grid was exposed, the plexiglass wall was up and a mild anise scent was used as an olfactory cue. The door to the sound dampening enclosure was shut for the duration of the trial. 180 s after the mice were placed in the training context, they were delivered a single footshock of 0.75 mA lasting 2 s. 15 s after the end of the footshock, the mice were placed back into their home cages. Non-alcoholic antiseptic wipes were used to clean the grids and catch trays in between trials. An hour later the mice were brought into the room in paper buckets and put into similar context B. The house fan and house light were turned off, the door of the enclosure was left ajar, plastic placemat sheets were put into the shuttle box to make a high-walled circular enclosure, the plexiglass wall was left down and a mild lemon scent was used as the olfactory cue. The stainless steel grid, a salient feature of both contexts, remained exposed. The mice stayed in the chambers for 180 s without receiving a footshock and were placed back into their home cages. 70% ethanol was used to clean the grids and catch trays between trials. The discrimination ratio allowed for evaluation of discrimination between the two contexts and was computed as follows: A = freezing in context A, B = freezing in context B, and the discrimination ratio = A/(A + B). Larger values indicate better discrimination.

### Brain areas microdissection

Animals were sacrificed by cervical dissociation. Brains were quickly removed and put in ice-cold slurry of 0.9% NaCl. Three consecutive rostra-caudal sections from Bregma −0.82 mm to −5.00 mm (Franklin and Paxinos, 1997) were collected by using a Mouse Brain Matrice (RBMA-200C, World Precision Instruments Inc., USA) and transferred into RNA stabilization solution (RNAlater, Ambion^®^, Applied Biosystems, France). Hippocampus was then separated under the microscope. All samples were stored in RNAlater at −80 °C until RNA extraction. LPH and Decorin expression were used as markers to confirm the quality of dissection of tissues as described previously^45^.

### RNA extraction

Total RNA was isolated from frozen tissue samples using TRIzol (Invitrogen, Carlsbad, Calif.) and according to the manufacturer’s protocol. RNA quality was assessed using the Biophotometer (Eppendorf) and gel electrophoresis with the RNA LabChip^®^ 6000 Nano kit (Bioanalyzer^®^ 2000, Agilent Technologies). RNA quality cutoff values was OD ratio 260/230 > 1.6 nm and OD ratio 260/280 ratio >1.7 nm, and RIN (RNA Integrity Number) >7.0. 0.5 μg of total RNA was then reverse-transcribed and converted into double-stranded cDNA using the qScript cDNA Synthesis Kit (Quanta Biosciences, Gaithersburg, Maryland).

### Real-Time qPCR

In brief, small PCR products (70–160 base-pairs) were amplified in quadruplets on an BioRad real-time PCR machine (CFX-96), using universal PCR conditions (65–59 °C touch-down, followed by 35 cycles (15 s at 95 °C, 10 s at 59 °C and 10 s at 72 °C)). cDNA was amplified in 20 μl reactions (3 mm MgCl2, 200 nM dNTPs, 200 nM primers, 0.5 unit Platinum Taq DNA polymerase (Invitrogen, Carlsbad, CA, USA)). The primer position for genes of interest was decided after an alignement of the sequence using BLAT (http://genome.ucsc.edu/cgi-bin/hgBlat). Primers were then designed using Primer 3 Plus, and if applicable, one of the primer matched the sequence corresponding to the probeset of the gene. For each gene, 2 pairs of primers were first tested and the most robust pair were kept for qPCR analysis. Primer dimers were assessed by amplifying primers without cDNA. Primers were retained if they produced no primer dimers or nonspecific signal only after 35 cycles. Results were calculated as the geometric mean of relative intensities compared to three internal controls (ACTIN, GAPDH, PPIA).

### Statistical analysis

For the neurogenesis study, data are presented as means ± SEM for BrdU, Ki67 or DCX or BrdU^+^/DCX^+^/NeuN^−^, BrdU^+^/DCX^+^/NeuN^+^, or BrdU*+* /DCX^−^/NeuN^+^ labeling -positive cells (Figs 1 and 2). For the real-time qPCR experiments, data are presented as means average expression (in %) ±SEM (Fig. 3). For the context discrimination task that models “context discrimination”, data are presented as means ± SEM for freezing (%) or discrimination ratio (Fig. 4). All data were analyzed by one-way ANOVA or two-way ANOVA with repeated measures were applied to the data as appropriate. Significant main effects and/or interactions were followed by Fisher’s PLSD post hoc analyses. Discrimination ratio was analyzed following a planned comparison (Fig. 4I).

Differences were considered significant when P ≤ 0.05. All analyses were conducted using Prism 6.0 h software (GraphPad).

## Additional Information

**How to cite this article**: Guilloux, J.-P. *et al*. S 38093, a histamine H_3_ antagonist/inverse agonist, promotes hippocampal neurogenesis and improves context discrimination task in aged mice.. *Sci. Rep.* **7**, 42946; doi: 10.1038/srep42946 (2017).

**Publisher's note:** Springer Nature remains neutral with regard to jurisdictional claims in published maps and institutional affiliations.

## Supplementary Material

Supplementary Information

Supplementary Video

## Figures and Tables

**Table 1:** Effects of chronic S 38093 administration on neurogenesis in the dentate gyrus of hippocampus in the APPSWE mouse model of Alzheimer’s disease.

Genotype		APPSWE^WT^		APPSWE^Tg^	
Treatment		Vehicle	S38093 (3 mg/kg/d)	Vehicle	S38093 (3 mg/kg/d)
**Proliferation**					
	*Ki67*^+^ *cells*	1158.0 (±*102.9*)	1527.8* (±*173.5*)	1200.0 (±*1132.3*)	1588.0* (±*154.85*)
**Survival**					
	*BrdU*^+^ *cells*	487.8 (±*33.1*)	657.6** (±*72.9*)	538.6 (±*47.8*)	1168.8** (±*218.2*)
**Maturation**					
	*Total DCX*^+^ *cells*	3280.0 (±*361.2*)	4692.0* (±655.1)	3982.8 (±440.1)	5030.4* (±659.5)
	*Total DCX*^+^ *cells with tertiary dendrites*	937.3 (±*168.95*)	1849.2* (±*60.9*)	1526.4 (±*245.8*)	2187.6* (±*368.5*)
	*Maturation index*	0.27 (±*0.023*)	0.37** (±*0.019*)	0.36 (±*0.031*)	0.41** (±*0.027*)

The effects of chronic administration of S 38093 (3 mg/kg/day p.o., 28 days) on cell proliferation, survival and maturation were compared to those of vehicle (purified water) in APPSWE^WT^ and APPSWE^Tg^ mice. Cell proliferation and survival were expressed by the number of KI67^+^ and BrdU^+^ cells respectively. Maturation was represented by the total number of DCX^+^ cells, the number of DCX^+^ cells with tertiary dendrites and the maturation index of newborn granule cell. Data are expressed as mean ± SEM. A two-way ANOVA was applied to the data. A significant effect of treatment for all the different analyzed parameters was observed in both genotypes (*p < 0.05, **p < 0.01). Significant interaction for Survival parameter was followed by Fisher’s PLSD post hoc analysis. **p < 0.01 compared to the APPSWE^WT^/vehicle group and APPSWE^Tg^/vehicle group.
