# Supplementary material for: S 38093, a histamine H3 antagonist/inverse agonist, promotes hippocampal neurogenesis and improves context discrimination task in aged mice
Source: Sci Rep. 2017 Feb 20;7:42946. doi: 10.1038/srep42946 (PMC5317168; doi:10.1038/srep42946)
Supplement: Supplementary Information [file srep42946-s1.pdf]

SUPPLEMENTARY INFORMATION

S 38093, a histamine H_3 antagonist/inverse agonist, promotes hippocampal neurogenesis and improves context discrimination task in aged mice.

Short Title: Neurogenic and behavioral effects of S 38093

Authors: Jean-Philippe Guilloux[1,*], Benjamin A Samuels[2,*], Indira Mendez-David[1,*], Alice Hu[3], Marjorie Levinstein[3], Charlène Faye[1], Maryam Mekiri[1], Elisabeth Mocaer[4], Alain M Gardier[1], René Hen[3,5], Aurore Sors[4], Denis J David[1,§].

[1]CESP/UMR-S1178, Univ. Paris-Sud, Fac. Pharmacie, INSERM, Université Paris-Saclay, Chatenay-Malabry, France.; [2]Behavioral and Systems Neuroscience Area, Department of Psychology, Rutgers The State University of New Jersey, Piscataway, NJ, USA ; [3]Departments of Neuroscience and Psychiatry, Columbia University, New York, USA; [4]Pôle d'Innovation Thérapeutique Neuropsychiatrie Servier, Suresnes, France; [5]Department of Integrative Neuroscience, New York State Psychiatric Institute, New York, USA

*Authors equally contribute to this work

[§] To whom correspondence should be sent:

Pr Denis David
CESP, Univ. Paris-Sud, Fac Pharmacie,
Inserm, Université Paris-Saclay 5, rue Jean-Baptiste Clément
92290 Chatenay-Malabry
Tel: +33146835968
Fax: +33146835355
denis.david@u-psud.fr

Supplementary Table 1: Overall statistical analysis of the neurogenic effects of S 38093 in 129/SvEv Tac mice.

Supplementary Table 2: Overall statistical analysis of the neurogenic effects of S 38093 in 3-month old or 16-month old C57BL/6JRj mice.

Supplementary Table 3: Overall statistical analysis of the neurogenic effects of S 38093 in APPSWE^{WT} and APPSW^{Tg} mice.

Supplementary Table 4: Overall statistical analysis of the effects of S 38093 on dendritic arborization in APPSWE^{WT} and APPSW^{Tg} mice.

Supplementary Table 5: Overall statistical analysis of the effects of S 38093 on gene expression in 3-month old or 16-month old C57BL/6JRj mice.

Supplementary Table 6: Statistical analysis of the effects of aging and S 38093 on context discrimination in 3-month old or 16-month old C57BL/6JRj mice.

Supplementary Table 7: Statistical analysis of the effects of aging and S 38093 on discrimination ratio in 3-month old or 16-month old C57BL/6JRj mice.

Supplementary Figure 1: Timeline of experiments

Effects of chronic S 38093 administration on neurogenesis were tested in 3-month-old 129/SvEv Tac mice (**S1A**). BrdU (150 mg/kg, i.p.) was injected twice a day for 3 days before the start of treatment, or 2 hours before sacrifice of the animals, at the end of the treatment. Neurogenic effects of S 38093 (0.3 and 3 mg/kg/d) were compared to a 4-week fluoxetine (18 mg/kg/d).

Effects of aging and chronic S 38093 administration on neurogenesis were tested in 3 and 16-month-old C57BL/6JRj mice at the start of the experiment (**S1B**). BrdU (150 mg/kg, i.p.) was injected twice a day for 3 days before the start of treatment. Neurogenic effects of S 38093 (0.3, 1 and 3 mg/kg/d) were compared to a 4-week donepezil (0.3 or 1 mg/kg/d) treatment.

Neurogenic effects of chronic administration of S 38093 (3 mg/kg/d, 28 days) were tested in the APPSWE mouse model of Alzheimer's Disease (**S1C**). BrdU (150 mg/kg, i.p.) was injected twice a day for 3 days before the start of treatment.

Behavioral effects of aging or chronic S 38093 administration (0.3 and 3 mg/kg/d, 28 days) were tested in 16-month-old C57BL/6JRj mice (**S1D**). Pattern Discrimination protocol was performed after 4 weeks of chronic treatment, during day 29 to 36.

Supplementary Figure 2: Chronic S 38093 administration enhances adult hippocampal neurogenesis along the septotemporal axis of the dentate gyrus of young adult 129/SvEvTac mice.

The effects of chronic administration of S 38093 (0.3 and 3 mg/kg/day p.o., 28 days) and fluoxetine (18 mg/kg/day p.o., 28 days) on cell proliferation (**S2A, B**), survival (**S2C, D**) and maturation (**S2E, F**) were compared to those of vehicle (purified water) along the septotemporal axis of the hippocampus. Cell proliferation (**S2A, B**) and survival (**S2C, D**) were expressed by the number of $BrdU^+$ cells. Maturation was represented by the total number of DCX^+ cells with tertiary dendrites (**S2E, F**). Data are expressed as mean ± SEM. A one-way ANOVA was applied to the data followed by Fisher's PLSD post hoc analysis as appropriate. *$p<0.05$, **$p<0.01$ compared to vehicle (n=5-10 mice of 3 months old per group).

Supplementary Figure 3: Effects of chronic S 38093 administration on dendritic maturation in the dentate gyrus of the hippocampus in the APPSWE model of Alzheimer's disease

The effects of chronic treatment with S 38093 (3 mg/kg/d, 28 days) on dendritic complexity were compared to those of vehicle in $APPSWE^{WT}$ and $APPSWE^{Tg}$ mice. The effects of tested treatments on dendritic length (**S3A, C**) and the number of intersection (**S3B, D**) were measured (n=3 mice/group, 20 cells/mouse) using a Sholl analysis of DCX^+ neurons in $APPSWE^{WT}$ and $APPSWE^{Tg}$ mice. A two-way ANOVA with repeated measures was applied to the data. Significant main effects and/or interactions were followed by Fisher's PLSD post hoc analysis. *$p<0.05$, **$p<0.01$ and ***$p<0.001$ for effects of S 38093 compared to vehicle. Representative image and traces from Sholl

analyses of DCX^+ cells with tertiary branches after vehicle or chronic S 38093 in $APPSWE^{WT}$ and $APPSWE^{Tg}$ (S3E).

Supplementary Figure 4: Representative illustrations of doublecortin (DCX) immunoreactive positive cells (10x magnification) in the dentate gyrus after 4 weeks of treatment with vehicle (S4A, 4E), fluoxetine (18 mg/kg/d) (S4B, 4F), S 38093 (0.3 or 3 mg/kg/d) (S4C, D, 4G, H) along the dorso/ventral axis in adult 129/SvEv Tac mice. Scale bar = 100μm.

Supplementary Figure 5: Representative illustrations of confocal BrdU immunoreactive positive cells alone (S5A), colocalized with doublecortin (DCX) (S5B), colocalized with DCX and NeuN (S5C), or colocalized with NeuN (S5D) (63x magnification) in the dentate gyrus in 129/SvEv Tac mice.

Supplementary Figure 6: Representative illustrations of $Ki67^+$ (S6A, 6B) or doublecortin (DCX) (S6C, 6D) immunoreactive positive cells (10x magnification) in the dentate gyrus in 3-month and 16-month old C57BL/6JRj mice. Scale bar = 100μm.

Supplementary Figure 7: Representative illustrations of doublecortin (DCX) (S7C, 7D) immunoreactive positive cells (10x magnification) in the dentate gyrus in APPSWE^{WT} and APPSWE^{Tg} after 4 weeks of treatment with S 38093 (3 mg/kg/d). Scale bar = 100μm.

Supplemental movie 1: Reconstruction of z-stack of BrdU^{+} cells colocalized with DCX^{+} cells in the dentate gyrus in 129/SvEv Tac strain taken with a Zeiss LSM 510 confocal microscope (63x magnification) using Imaris 7.8.

Supplementary Figure 1

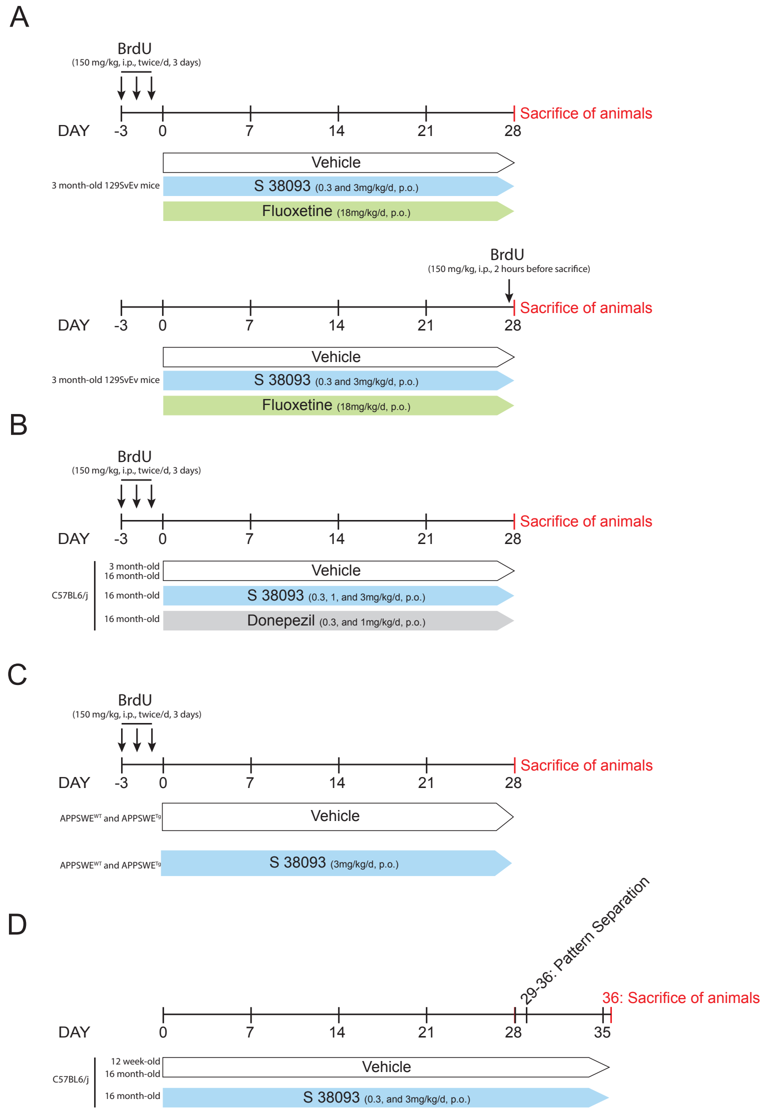

Supplementary Figure 2

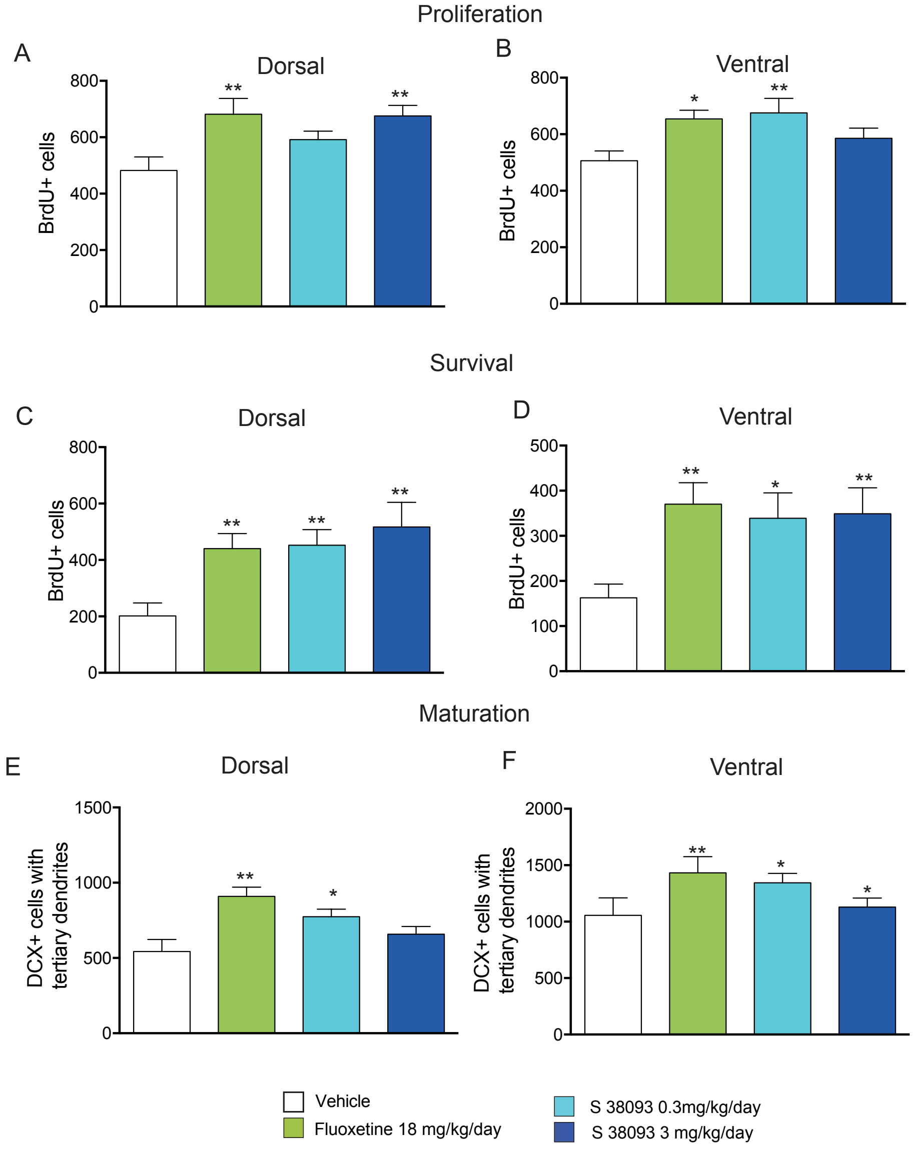

Supplementary Figure 3

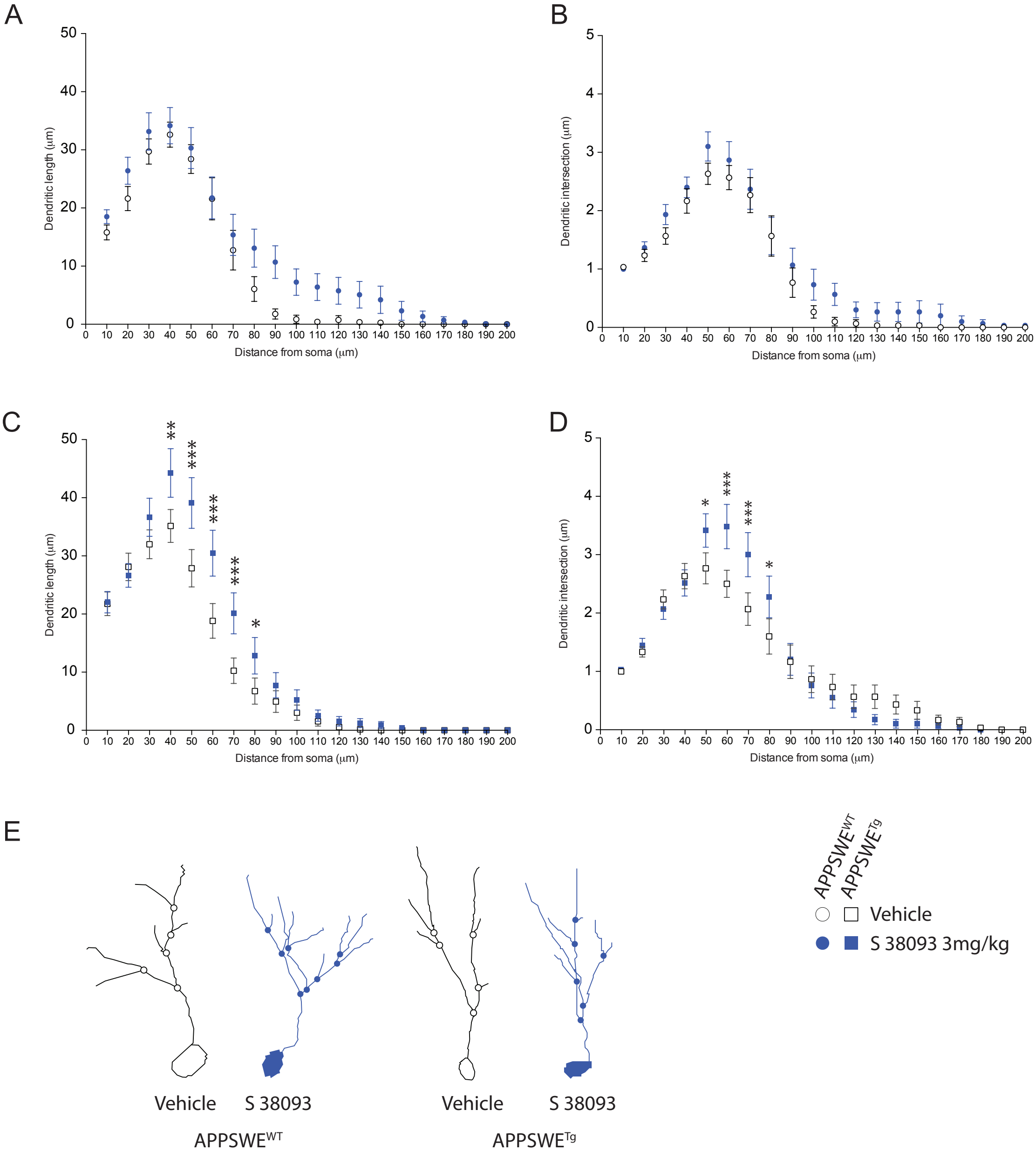

Supplemental Figure 4

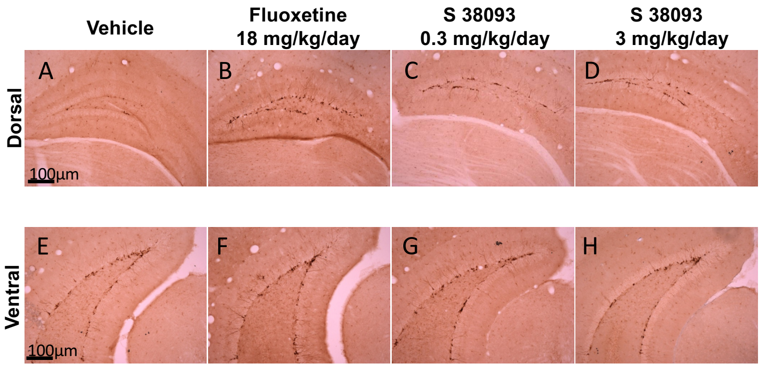

Supplemental Figure 5

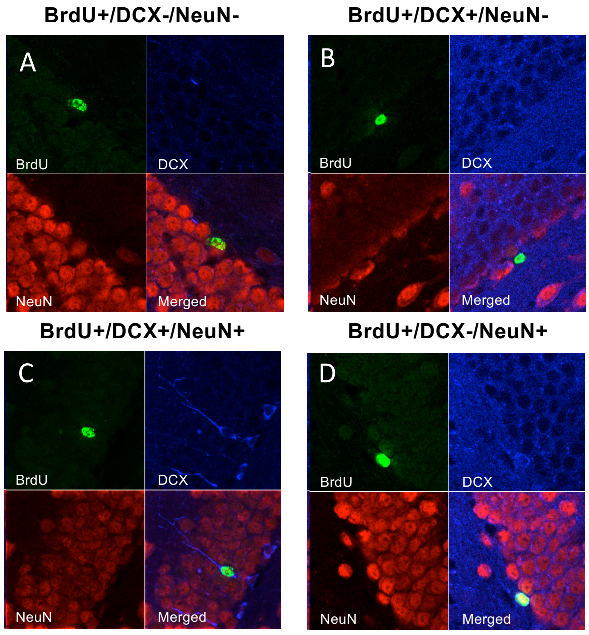

Supplemental Figure 6

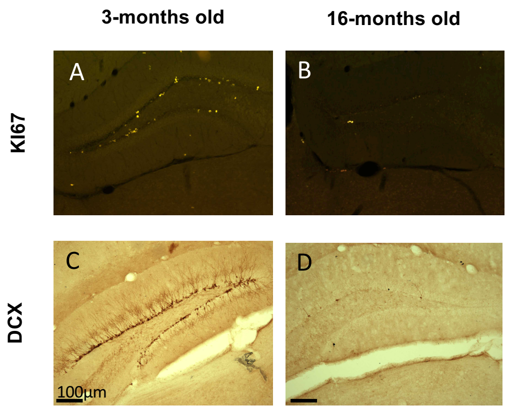

Supplemental Figure 7

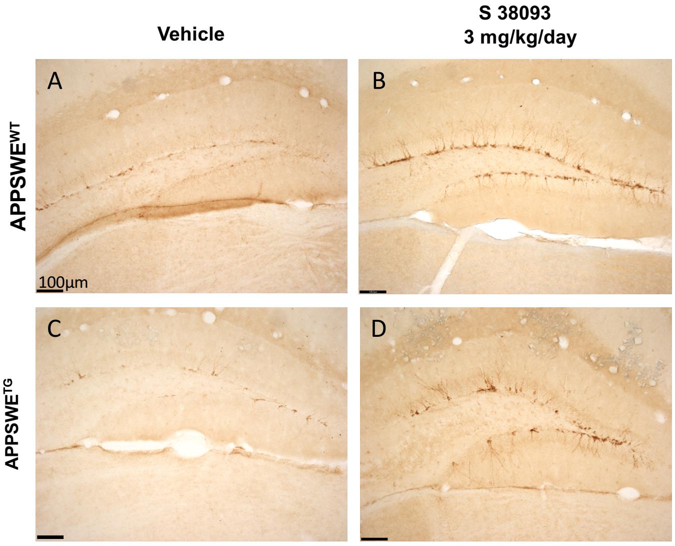

Supplementary Table 1: Overall statistical results

	Test	Parameter	F values	One-Way ANOVA F-value	One-Way ANOVA P-value	Fluox vs Veh	S38093 (0.3) vs Veh	S38093 (3) vs Veh	S38093 (0.3) vs Fluox	S38093 (3) vs Fluox
**Figure 1**										
**1A**	Proliferation	BrdU positive cells (proliferation)	3, 30	5.429	0.0073	0.0036	0.0827	0.0021	0.1761	0.9253
**1B**	Survival	BrdU positive cells (survival)	3, 31	4.691	0.0082	0.0077	0.0054	0.0017	0.8803	0.3866
**1C**	Maturation	Total DCX Cells	3, 22	1.656	0.2055					
**1D**	Maturation	DCX cells with tertiary dendrites	3, 22	4.119	0.0184	0.0047	0.0246	0.3907	0.3536	0.0281
**1E**	Maturation	Maturation Index	3, 22	5.879	0.0042	0.0005	0.0119	0.0188	0.1346	0.096
**1F**	ell differentiati	Relative proportion of BrdU+/NeuN+	3, 15	5.109	0.0124	0.0016	0.0203	0.0732	0.2046	0.0598
**Supplemental figure S2**										
**S2A**	Proliferation Dorsal	BrdU positive cells (proliferation)	3, 30	4.835	0.0073	0.0036	0.0827	0.0021	0.1761	0.9253
**S2B**	Proliferation Ventral	BrdU positive cells (proliferation)	3, 30	3.777	0.0207	0.0154	0.0047	0.1417	0.7145	0.2321
**S2C**	Survival Dorsal	BrdU positive cells (survival)	3, 31	4.840	0.0071	0.0077	0.0054	0.0017	0.8803	0.3866
**S2D**	Survival Ventral	BrdU positive cells (survival)	3, 31	3.4778	0.0276	0.0059	0.0175	0.0211	0.6408	0.7718
**S2E**	Maturation Dorsal	DCX cells with tertiary dendrites	3,22	5.867	0.0042	0.0007	0.0121	0.1885	0.1603	0.0128
**S2F**	Maturation Ventral	DCX cells with tertiary dendrites	3, 31	3.4778	0.0276	0.0059	0.0175	0.0211	0.6408	0.7718

Supplementary Table 2: Overall statistical results

	Test	Parameter	F values	One-Way ANOVA F-value	One-Way ANOVA P-value	Donepezil (0.3) vs Old Veh	Donepezil (1) vs Old Veh	S38093 (0.3) vs Old Veh	S38093 (1) vs Old Veh	S38093 (3) vs Old Veh	S38093 (0.3) vs Donepezil (0.3)	S38093 (1) vs Donepezil (0.3)	S38093 (3) vs Donepezil (0.3)	S38093 (0.3) vs Donepezil (1)	S38093 (1) vs Donepezil (1)	S38093 (3) vs Donepezil (1)	Old Veh vs Young Veh (Student T-Test)
**Figure 2**																	
**2A**	Proliferation	Ki67 positive cells (proliferation)	5, 20	3.183	0.0283	0.1504	0.1321	0.0242	0.0048	0.0037	0.276	0.1049	0.1012	0.4612	0.2352	0.2411	<0.0001
**2B**	Survival	BrdU positive cells (survival)	5, 32	4.545	0.003	0.9674	0.0839	0.2033	0.0185	0.0002	0.2842	0.0531	0.0016	0.8394	0.5046	0.0258	<0.0001
**2C**	Maturation	Total DCX Cells	5, 28	3.223	0.0201	0.4252	0.1838	0.655	0.6215	0.0215	0.3025	0.8402	0.0049	0.1518	0.5105	0.0012	<0.0001
**2D**	Maturation	DCX cells with tertiary dendrites	5, 28	6.66	0.0003	0.8706	0.3128	0.4766	0.957	0.0002	0.4211	0.9321	0.0002	0.1511	0.4257	< 0.0001	<0.0001
**2E**	Maturation	Maturation Index	5, 28	3.44	0.0151	0.5224	0.8111	0.4629	0.6809	0.0014	0.8291	0.8836	0.0126	0.3774	0.5558	0.0015	<0.0001
**2F**	Cell differentiation	Relative proportion of BrdU+/NeuN+ cells	5, 22	2.752	0.046	0.2764	0.9898	0.8237	0.0536	0.0408	0.2034	0.0088	0.0105	0.8336	0.055	0.0622	<0.0001

Supplementary Table 3: Overall statistical results

	**Test**	**Parameter**	**F values**	**Two-Way ANOVA F-value Genotype**	**Two-Way ANOVA P-value Genotype**	**Two-Way ANOVA F-value Treatment**	**Two-Way ANOVA P-value Treatment**	**Two-Way ANOVA F-value Interaction**	**Two-Way ANOVA P-value Interaction**	**APPSWE-WT: S38093 (3) vs Veh**	**APPSWE-Tg: S38093 (3) vs Veh**
**Table 1**											
**APPSWE-WT/APPSWE-Tg**	Proliferation	Ki67 positive cells (proliferation)	1, 34	0.1200	0.7312	6.568	0.015	0.003874	0.950		
**APPSWE-WT/APPSWE-Tg**	Survival	BrdU positive cells (survival)	2, 30	10.94	0.0024	22.16	0.0001	7.339	0.011	0.12	<0.0001
**APPSWE-WT/APPSWE-Tg**	Maturation	Total DCX Cells	2, 35	0.8823	0.3540	4.923	0.0331	0.1081	0.7443		
**APPSWE-WT/APPSWE-Tg**	Maturation	DCX cells with tertiary dendrites	2, 35	2.329	0.136	6.700	0.0139	0.1701	0.6825		
**APPSWE-WT/APPSWE-Tg**	Maturation	Maturation Index	2, 35	5.92	0.0202	9.164	0.0046	0.9852	0.3277		

Supplementary Table 4: Overall statistical results

	Test	Parameter	F values	Two-Way ANOVA with repeated measure F-value (length or intersection effect)	Two-Way ANOVA with repeated measure p-value (length or intersection effect)	F values	Two-Way ANOVA with repeated measure F-value (Distance effect)	Two-Way ANOVA with repeated measure p-value (Distance effect)	F values	Two-Way ANOVA with repeated measure F-value (Interaction)	Two-Way ANOVA with repeated measure p-value (Interaction)	APPSWE-WT : S38093 vs Vehicle	APPSWE-KO : S38093 vs Vehicle
**Supplementary Figure 3A**													
**3A**	Maturation	Dendritic Length in WT	1, 58	6.407	0.0141	19, 1102	76.53	<0.0001	19, 1102	0.9388	0.5338		
**3A**	Maturation	Dendritic Length at 10µm											
**3A**	Maturation	Dendritic Length at 20µm											
**3A**	Maturation	Dendritic Length at 30µm											
**3A**	Maturation	Dendritic Length at 40µm											
**3A**	Maturation	Dendritic Length at 50µm											
**3A**	Maturation	Dendritic Length at 60µm											
**3A**	Maturation	Dendritic Length at 70µm											
**3A**	Maturation	Dendritic Length at 80µm											
**3A**	Maturation	Dendritic Length at 90µm											
**3A**	Maturation	Dendritic Length at 100µm											
**3A**	Maturation	Dendritic Length at 110µm											
**3A**	Maturation	Dendritic Length at 120µm											
**3A**	Maturation	Dendritic Length at 130µm											
**3A**	Maturation	Dendritic Length at 140µm											
**3A**	Maturation	Dendritic Length at 150µm											
**3A**	Maturation	Dendritic Length at 160µm											
**3A**	Maturation	Dendritic Length at 170µm											
**3A**	Maturation	Dendritic Length at 180µm											
**3A**	Maturation	Dendritic Length at 190µm											
**3A**	Maturation	Dendritic Length at 200µm											
**Supplementary Figure 3B**													
**3B**	Maturation	Dendritic Intersection in WT	1, 58	3.201	0.0788	19, 1102	75.89	<0.0001	19, 1102	0.4623	0.9764		
**3B**	Maturation	Dendritic Intersection at 10µm											
**3B**	Maturation	Dendritic Intersection at 20µm											
**3B**	Maturation	Dendritic Intersection at 30µm											
**3B**	Maturation	Dendritic Intersection at 40µm											
**3B**	Maturation	Dendritic Intersection at 50µm											
**3B**	Maturation	Dendritic Intersection at 60µm											
**3B**	Maturation	Dendritic Intersection at 70µm											
**3B**	Maturation	Dendritic Intersection at 80µm											
**3B**	Maturation	Dendritic Intersection at 90µm											
**3B**	Maturation	Dendritic Intersection at 100µm											
**3B**	Maturation	Dendritic Intersection at 110µm											
**3B**	Maturation	Dendritic Intersection at 120µm											
**3B**	Maturation	Dendritic Intersection at 130µm											
**3B**	Maturation	Dendritic Intersection at 140µm											
**3B**	Maturation	Dendritic Intersection at 150µm											
**3B**	Maturation	Dendritic Intersection at 160µm											
**3B**	Maturation	Dendritic Intersection at 170µm											
**3B**	Maturation	Dendritic Intersection at 180µm											
**3B**	Maturation	Dendritic Intersection at 190µm											
**3B**	Maturation	Dendritic Intersection at 200µm											
**Supplementary Figure 2C**													
**2C**	Maturation	Dendritic Length in Tg	1, 58	7.553	0.0080	19, 1102	105.7	<0.0001	19, 1102	2.426	0.0006		
**2C**	Maturation	Dendritic Length at 10µm											0.9246
**2C**	Maturation	Dendritic Length at 20µm											0.6095
**2C**	Maturation	Dendritic Length at 30µm											0.1037
**2C**	Maturation	Dendritic Length at 40µm											0.0014
**2C**	Maturation	Dendritic Length at 50µm											< 0.0001
**2C**	Maturation	Dendritic Length at 60µm											< 0.0001
**2C**	Maturation	Dendritic Length at 70µm											0.0005
**2C**	Maturation	Dendritic Length at 80µm											0.0332
**2C**	Maturation	Dendritic Length at 90µm											0.3252
**2C**	Maturation	Dendritic Length at 100µm											0.451
**2C**	Maturation	Dendritic Length at 110µm											0.7285
**2C**	Maturation	Dendritic Length at 120µm											0.7259
**2C**	Maturation	Dendritic Length at 130µm											0.7101
**2C**	Maturation	Dendritic Length at 140µm											0.7594
**2C**	Maturation	Dendritic Length at 150µm											0.8903
**2C**	Maturation	Dendritic Length at 160µm											0.9888
**2C**	Maturation	Dendritic Length at 170µm											> 0.9999
**2C**	Maturation	Dendritic Length at 180µm											> 0.9999
**2C**	Maturation	Dendritic Length at 190µm											> 0.9999
**2C**	Maturation	Dendritic Length at 200µm											> 0.9999
**Supplementary Figure 2D**													
**2D**	Maturation	Dendritic Intersection in Tg	1, 58	3.614	<0.0001	19, 1102	70.05	<0.0001	19, 1102	2.467	0.0005		
**2D**	Maturation	Dendritic Intersection at 10µm											0.8996
**2D**	Maturation	Dendritic Intersection at 20µm											0.6741
**2D**	Maturation	Dendritic Intersection at 30µm											0.5476
**2D**	Maturation	Dendritic Intersection at 40µm											0.671
**2D**	Maturation	Dendritic Intersection at 50µm											0.018
**2D**	Maturation	Dendritic Intersection at 60µm											0.0003
**2D**	Maturation	Dendritic Intersection at 70µm											0.0007
**2D**	Maturation	Dendritic Intersection at 80µm											0.0135
**2D**	Maturation	Dendritic Intersection at 90µm											0.883
**2D**	Maturation	Dendritic Intersection at 100µm											0.6926
**2D**	Maturation	Dendritic Intersection at 110µm											0.5064
**2D**	Maturation	Dendritic Intersection at 120µm											0.4171
**2D**	Maturation	Dendritic Intersection at 130µm											0.1494
**2D**	Maturation	Dendritic Intersection at 140µm											0.2276
**2D**	Maturation	Dendritic Intersection at 150µm											0.4004
**2D**	Maturation	Dendritic Intersection at 160µm											0.7208
**2D**	Maturation	Dendritic Intersection at 170µm											0.7176
**2D**	Maturation	Dendritic Intersection at 180µm											0.9029
**2D**	Maturation	Dendritic Intersection at 190µm											> 0.9999
**2D**	Maturation	Dendritic Intersection at 200µm											> 0.9999

Supplementary Table 5: Overall statistical results

	Test	Parameter	F values	One-Way ANOVA F-value	One-Way ANOVA P-value	Young Veh vs Old Veh	Donepezil (0.3) vs Old Veh	Donepezil (1) vs Old Veh	S38093 (0.3) vs Old Veh	S38093 (1) vs Old Veh	S38093 (3) vs Old Veh
**Figure 3**											
**3A**	BDNF exon I	Average expression level	6, 35	3.464	0.0086	0.004	0.3246	0.3421	0.1156	0.0053	0.0014
**3B**	BDNF exon IV	Average expression level	6, 44	4.571	0.0011	0.0208	0.3401	0.999	0.0758	0.8695	0.0208
**3C**	BDNF exon VI	Average expression level	6, 45	0.7969	0.5788						
**3D**	BDNF exon IX	Average expression level	6, 44	6.559	<0.0001	0.0134	0.0237	0.1745	0.8548	0.0325	0.0134
**3E**	VEGFA	Average expression level	6, 44	2.049	0.0791						
**3F**	IGF1	Average expression level	6, 44	1.502	0.1999						
**3G**	H3 receptor	Average expression level	2, 13	0.07371	0.9293	0.8276				0.8616	0.7079

Supplementary Table 6: Overall statistical results

	Test	Parameter	Treatment groups number	Total number of samples	Two-Way ANOVA F-value Aging or Treatment	Two-Way ANOVA P-value Aging or Treatment	Treatment groups number	Total number of samples	Two-Way ANOVA F-value Time	Two-Way ANOVA P-value Time	Treatment groups number	Total number of samples	Two-Way ANOVA F-value Interaction	Two-Way ANOVA P-value Interaction	Day 3: Context A vs Context B	Day 4: Context A vs Context B	Day 5: Context A vs Context B	Day 6: Context A vs Context B	Day 7: Context A vs Context B	Day 8: Context A vs Context B	Day 9: Context A vs Context B	Day 10: Context A vs Context B	Day 11: Context A vs Context B	Student T-test p-value at
**Figure 4**																								
**4B**	Contextual Fear Conditionning Young	Freezing (%)	1	14	125.0	<0.0001	9	126	4.190	<0.0001	9	126	3.948	0.0002	<0.0001	<0.0001	<0.0001	<0.0001	<0.0001	<0.0001	<0.0001	<0.0001	< 0,0001	< 0,0001
**4C**	Contextual Fear Conditionning Old	Freezing (%)	1	14	25.82	0.0002	9	126	5.913	<0.0001	9	126	4.768	<0.0001	0,6383	0,1247	0,0322	0,0208	0,0221	0,0629	0,1119	0,0666	< 0,0001	< 0,0001
**4D**	Contextual Fear Conditionning	Discrimination Ratio	2	26	14.35	<0.0001	10	260	1.969	0.0369	10	260	2.179	0.0031	0.2147	0.0131	<0.0001	0.0016	<0.0001	0.0349	<0.0001	<0.0001	0.0004	0.0003
**4F**	Contextual Fear Conditionning Vehicle	Freezing (%)	1	11	16.41	0.0019	5	55	0.9565	0.4524	5	55	3.5000	0.0081	0,6813	0,8945	0,8606	0,673	0,0019	< 0,0001				
**4G**	Contextual Fear Conditionning Low Dose	Freezing (%)	1	12	34.96	<0.0001	5,0000	60,0000	0.8362	0.5293	5	60	5.880	0.0002	0,5226	0,2222	0,0013	0,0086	< 0,0001	< 0,0001				
**4H**	Contextual Fear Conditionning High Dose	Freezing (%)	1	13	23.35	0.0003	5,0000	65,0000	0.7400	0.5962	5	65	1.075	0.3826										

Supplementary Table 7: Overall statistical results

	Test	Parameter	Treatment groups number	Total number of samples	Two-Way ANOVA F-value Aging or Treatment	Two-Way ANOVA P-value Aging or Treatment	Treatment groups number	Total number of samples	Two-Way ANOVA F-value Time	Two-Way ANOVA P-value Time	Treatment groups number	Total number of samples	Two-Way ANOVA F-value Interaction	Two-Way ANOVA P-value Interaction	Day 3: planned comparison	Day 4: planned comparison	Day 5: planned comparison	Day 6: planned comparison	Day 7: planned comparison	Day 8: planned comparison
**Figure 4**																				
**4I**	Contextual Fear Conditionning	Discrimination Ratio	2	36	2.107	0.1363	5,0000	180,0000	7.277	<0.0001	10	180	1.041	0.4113	*0.4658*	*0.3968*	*0.0372*	*0.1183*	*0.1715*	*0.7978*
